# Supplementary material for: Socio-demographic factors associated with normal linear growth among pre-school children living in better-off households: A multi-country analysis of nationally representative data
Source: PLoS One. 2020 Mar 11;15(3):e0224118. doi: 10.1371/journal.pone.0224118 (PMC7065827; doi:10.1371/journal.pone.0224118)
Supplement: Attachment — Submitted filename: Reviewers_Comments.docx [file pone.0224118.s001.docx]

PONE-D-19-27838
Positive and negative determinants of normal linear growth among pre-school children living in better-off households: an analysis of secondary data from sub-Saharan Africa
PLOS ONE

**Comments:** This is an interesting manuscript. Both well-written reviews of it note some merit in this manuscript, but have highlighted a number of areas where it needs improvement. I agree that it is very important to state why data from these 5 specific countries were used and why only data from children in higher income families were analysed. Both reviewers highlight the need to add some additional potential confounders to the statistical models, which I think is of key importance. I also think the influence of paternal factors on childhood linear growth merits further thought in a revised manuscript. Although these are the key points, all the limitations highlighted by the reviewers require dealing with in a revised manuscript.

Response: We thank you for these positive comments. Please, find below our responses to the reviewers comments. Regarding why we used 5 countries, the following statement is captured in the manuscript “The selection of these five countries was informed by our previous work (12, 32)”

Journal Requirements:

When submitting your revision, we need you to address these additional requirements.

**Comment: 1.** Please ensure that your manuscript meets PLOS ONE's style requirements, including those for file naming. The PLOS ONE style templates can be found at

http://www.journals.plos.org/plosone/s/file?id=wjVg/PLOSOne_formatting_sample_main_body.pdf and <http://www.journals.plos.org/plosone/s/file?id=ba62/PLOSOne_formatting_sample_title_authors_affiliations.pdf>

Response: This now been done

**Comment 2:** We noticed you have some minor occurrence of overlapping text with the following previous publication(s), which needs to be addressed:

- Amugsi DA, Dimbuene ZT, Kimani-Murage EW, Mberu B, Ezeh AC. Differential effects of dietary diversity and maternal characteristics on linear growth of children aged 6-59 months in sub-Saharan  Africa: a multi-country analysis. Public health nutrition. 2017;20(6):1029-45

The text that needs to be addressed involves some paragraphs of the Introduction and of the Discussion.

In your revision ensure you cite all your sources (including your own works), and quote or rephrase any duplicated text outside the methods section. Further consideration is dependent on these concerns being addressed.

Response: This has now been addressed

**Comment 3:** We suggest you thoroughly copyedit your manuscript for language usage, spelling, and grammar. If you do not know anyone who can help you do this, you may wish to consider employing a professional scientific editing service.  

Response: This has been done. The paper was carefully edited using Grammarly (Premium version).

Reviewer #1:

**Comment:** Overall, you have presented an interesting manuscript of public health importance in sub-Saharan African countries. Reducing child growth faltering and ensuring optimal child growth remains a priority public health agenda among most developing countries in Africa and beyond. The study would serve as an additional input in nutrition policy makings. Your use of data from DHS as well as the inclusion of 5 countries increases the reliability and generalizability of the findings. Your analysis methodology, logistic regression, fits the objective of the study and the dichotomized nature of the outcome variable. The sections of the manuscript, introduction to conclusion, are presented in a clear and understandable way.

However, there are some major and minor issues that need to be addressed before the manuscript is accepted for publication.

Response: We thank the reviewer for these positive comments

Major comments (Methodology)

**Comment:** Title: Consider restating the title. For example, “Factors associated with optimal growth status of under-5 children in….”

Response: The title now reads “Factors associated with normal linear growth among pre-school children living in better-off households: a multi-country analysis of nationally representative data”

**Comment:** Study population: It is not clear why you restricted the study to only the better-off households, given growth faltering is mainly the problem of the poor, not the rich. Please clarify it.

Response: The paper is not focusing on growth flattering (stunted children) but on children who are growing well (non-stunted). This sets this paper apart from the usual practices of focusing on children who are not growing well (growth flattering) and the risk and protective factors of the same. We limited the analysis to better-off households in order to elucidate the factors that make children in these households to have better health outcomes relative to children in poor households. This has already been explained in the manuscript.

**Comment:** In all DHS surveys, the samples are collected following a two-stage cluster sampling scheme. Besides, the DHS sampling scheme over-represents small regions (sub-national divisions). Thus, all analyses, descriptive as well as regression, should take into account the cluster design of the study and be done after weighting the data by the corresponding weighting variable (i.e. v005 in the DHS datasets). Regression outputs of the weighted data would be surely different from the regression outputs of unweighted data for the same measure. Otherwise, the estimates would be biased towards the over-represented states. At the current state, the manuscript doesn’t provide any information on how these issues were handled. Thus, I strongly recommend rerunning the data on the weighted sample and taking into account the design effect of the cluster sampling.

*Response*: This was done but not explicitly captured in the first version of the manuscript. We have now added this sentence to address the concern “Because the DHS utilised complex sample design, we accounted for design effect in all the analysis using parametres such as primary sampling unit, strata and weight”.

**Comment:** Most of the variables with a proven influence on growth are not included in the analysis. For example, frequency of feeding, quality of complementary feeding, early initiation of breastfeeding, exclusive breastfeeding, vitamin A supplementation, history of infection, hygiene, sanitation, health care utilization, media exposure...etc. You included only dietary diversity variables. I am aware that these variables are included in the DHS datasets you used. What is your rationale to exclude these variables?

*Response:* We thank the reviewer for this comment. You are right, these variables are contained in the DHS data sets. However, to avoid making the results difficult to interpret and the tables unnecessary long (they are already long) or congested, after identifying the potential drivers (including the above referenced variables) of child growth per the literature and the UNICEF conceptual framework, we subjected them to bivariate analysis and included only statistical significant variables in the multivariable analysis. This is a standard practice in statistical analysis, since the researcher cannot include every potential factor in statistical models. Our own previous work and many others have shown that some the above referenced “proven” variables did not have a statistical significant relationship with child growth/health outcomes.

**Comment:** The measurement of the predictor variables is not provided and need to be included during your revision. For example, I could not understand what the variable “breastfeeding’ refers to. It is not clear whether it refers to the duration of breastfeeding, exclusive breastfeeding or early initiation of breastfeeding. How you developed the dietary diversity scores is also not clear. I suggest including a separate section for ‘variables definition and measurement’.

Response: We thank the reviewer for this observation. We have now included a separate section in the manuscript titled “predictor variables”. See the paragraph below:

We classified the predictor variables into child (dietary diversity, age and sex), maternal (body mass index, education, age, work status, parity, breastfeeding status, marital status, antenatal attendance), household (sex of household head, household size, number of children under 5 years) and community (place of residence) level variables. The child dietary diversity (DD) was created by counting the number of food groups the mother reported the child had consumed in the past 24h before the interview. In accordance with recommended procedures on the construction of the child DD indicator (42), we regrouped the food types in the data into seven (7) main categories (12): (i) grains, roots and tubers; (ii) legumes and nuts; (iii) dairy products; (iv) ﬂesh foods and organ meats; (v) vitamin A-rich fruits and vegetables; (vi) eggs; and (vii) other fruits and vegetables. A value of 1 was given for the child’s consumption of any of the food groups within 24h, while we assigned 0 for non-consumption (12). These scores were then summed up to obtain the DD score, ranging from 0 to 7, and used in the analysis as a categorical predictor variable. Also, a score of 1 was assigned to a mother who indicated she was still breastfeeding, and 0 for “no” response. Marital status was recoded into “not in union” “cohabiting” and “married”. Maternal body mass index (BMI), also referred to as Quetelet’s Index (43), was derived by dividing weight in kilograms by the squared height in meters (12). The BMI (kg/m^2^) was then classified into BMI<18.50 kg/m^2^ (underweight), BMI=18.50-24.99 kg/m^2^ (normal weight), BMI=25.0-29.9 kg/m^2^ (overweight) and BMI≥ 30.0 kg/m^2^ (obese) (44). We used BMI<18.50 kg/m^2^ (underweight) as a referenced category in the analysis. We selected the predictor variables based on the UNICEF conceptual framework of childcare (9) and the literature. We subjected the selected variables to bivariate analysis to establish their relationship with the outcome variable. Only statistically significant variables were included in the multivariable analysis.

**Comment:** As far as I know DHS datasets, some of the variables you used are only available for children below 24 months of age. For example, the dataset doesn’t enable to develop dietary diversity score for those above 24 months. Given your samples are all under-5 children, it is not clear how you handle this issue. Please explain.

Response: We thank the reviewer for the comment. This point is not entirely accurate. The DHS collects data on dietary intake of children up to 59 months old. These data are then used by analyst to construct the dietary diversity score. We have used this variable either as an outcome or predictor variable in not less than 15 scientific papers.

**Comment:** A problem with DHS datasets is data missing. What was the level of data missing in your case? How you managed the data missing? Have you checked whether the missing cases were not different from the included cases? Please Explain. Additionally, it would be more informative to include a flow of chart of your ample selection process.

Response: This point is not also entirely accurate. Although the DHS data may have some issues with missing data, these issues are addressed before the data are made available to users as the following statement on their website suggests “One of the primary goals of The DHS Program is to produce high-quality data and make it available for analysis in a coherent and consistent form. However, national surveys in developing countries are prone to incomplete or partial reporting of responses. Additionally, complex questionnaires inevitably allow scope for inconsistent responses to be recorded for different questions. For the analyst this results in a data file containing incomplete or inconsistent data, complicating the analysis considerably. In order to avoid these problems, The DHS Program has adopted a policy of editing and imputation which results in a data file that accurately reflects the population studied and may be readily used for analysis. Primary data quality policies include”: (<https://dhsprogram.com/data/Data-Quality-and-Use.cfm>).

We have been using the DHS data for several years and missing data have never been one of the issues we worry about.

**Comment:** You mentioned ‘sub-Saharan countries’ in the title but you used the data of only five countries. The selection of the five countries is not clear.

Response: The title now reads “Factors associated with normal linear growth among pre-school children living in better-off households: a multi-country analysis of nationally representative data”


Minor comments
**Comment:** Line 110-111: the statement ‘Statistical analyses that simultaneously examine the positive as well as negative determinants of normal child growth are missing’ is not right. There are various studies, including a study of mine, that examined the determinants of child growth simultaneously. Better to tone down the statement.

Response: We have changed “missing” to limited. Nevertheless, it is important to point out that the analytical strategy used in this paper is somewhat different from what is common in the literature. Instead of focusing on growth flattering (stunting) and the associated risk and protective factors, we focused on normal child growth (non-stunted children) and the factors that explain why these children are not stunted. We will like to invite the reviewer (s) to look at the paper from this angle.

**Comment:** Lines 188-189: You recoded middle, richer and richest households into “better-off households”. What is your rationale? Please provide a reference.

Response: We thank the reviewer for this comment. The literature on the positive health outcomes of children living in the middle to richest households is abound. What this study seeks to do is to understand the key factors that make children in these households to have better health outcomes relative to those living in poor households.

**Comment:** Table 1: Check the heading for consistency (raw 2 in particular).

Response: This has been corrected

**Comment:** Table 1: The SD columns are empty for the categorical variables, while SD values are provided for the variables on a continuous scale. Why not for the categorical variables? Besides, instead of SD, I would generally suggest presenting the 95%CI of the prevalence or means values of each variable.

Response: We thank reviewer for this comment. We feel that it will not make scientific sense to present mean estimates for categorical variables. Also, Table 1 presents basic descriptive analysis, therefore, no confidence intervals are required.

**Comment:** Lines 259: put space after 1.71 (aOR=1.75, 95% CI=1.17,2.62). Please check the whole manuscript for errors like this.

Response: This has been done

**Comment:** Line 310: “The results shed new insights on both positive and negative determinants of child growth….” As your findings are not new for those in the field, please paraphrase the statement.

Response: This concern has been addressed

**Comment:** Lines 325-326: How do you explain the unexpected finding that education was not significantly associated with growth in Kenya?

Response: We thank the reviewer for this observation. This is really not an unexpected findings. There are several studies (including ours), which did not show statistical significant effect of education on child health outcomes. Thus, it is not the case that education always has statistical significant effect on child health.

**Comment:** Lines 342-343: Again how do you explain the null association between breastfeeding and growth in two of the five countries?

Response: We thank the reviewer for this comment. Breastfeeding is critical for positive child health outcomes, but it is not always the case that its effects would be statistically significant. The literature on the null association between breastfeeding and child growth outcomes is abound (we did show this in the manuscript). Indeed, there are several other studies (including ours) that showed inverse relationship between breastfeeding and child growth…the concept of reverse causality.

**Comment:** Lines 422-423: “The novelty of this study is its focus on positive child growth outcomes”. This seems exaggerated given there are various studies that examined the determinants of child growth.

Response: We thank the reviewer for this comment. However, we respectfully disagree that this is an exaggeration. We have published several papers on childhood stunting (growth flattering) but this is the first time we are focusing on non-stunted children. This is also case in the literature. We will like to invite the reviewers to take note of the fact that in this paper, the outcome of interest is not stunted children but non-stunted.

**Comment:** There are editorial, punctuation and formatting errors. Example Line 76: the statement ‘Implementation of these programs more often than not places more emphasis on child…” Please check the manuscript thoroughly.

Response: We thank the reviewer for this observation. The manuscript has been thoroughly checked using grammarly (premium version)

Reviewer #2: Abstract

**Comment:** Sentence 5: Higher maternal weight (measured by body mass index) ◊ should it be maternal adiposity (?)

Response: It is maternal weight (i.e. BMI)

**Comment:** aOR is presented with CI, please also provide with p values

Response: We thank the reviewer for this comment. We felt that presenting both confidence intervals and p-values will make the results difficult to read/congested. For statistical significance, the CIs are considered more robust than p-values, in our view. However, the level of statistical significance (p-values) has been presented at the end of every table (except Table 1, in which we did not test any statistical significance).

Introduction

Comments about language/writing style:

**Comment: 1.** I would suggest improving the writing with more academic and indirect style (eg Line 65: “for children’s sake”, line 208: “we did this”, etc)

Response: This has been corrected

**Comment: 2.** I would suggest consistency in the type of English pronunciation used, which I suppose should be American English (eg Line 76: programmes ◊ programs)

Response: We thank the reviewer for this comment. The manuscript is written in British English. We have carefully revised the manuscript using grammarly premium to ensure consistency.

Comments about content/analysis in general:

**Comment:** The paper addresses an important issue in public health by showing data on the child’s growth in LMIC with large number of subjects. While the topic is very exciting, LMIC portrays some complicated issues, relating to infections, poverty, lifestyle, and other morbidities.

Response: We thank the reviewer for this positive comment

**comment: 1.** There are some more factors that need to be considered in looking at children’s linear growth in LMIC. Checking at the DHS report, these data are available thus need to be included in the analyses: infections (eg malaria, TB, HIV both among children and parents), delivery method/assistance, parental tobacco smoking, sanitation, vaccination, and other morbidities (especially anaemia)

Response: We thank the reviewer for this comment. You are right, these variables are contained in the DHS data sets, although some of them may be country specific. However, to avoid making the results difficult to interpret and the tables unnecessary long (they are already long) or congested, after we’ve identified the potential drivers of child growth per the literature and the UNICEF conceptual framework, we subjected them to bivariate analysis and included only statistical significant variables in the multivariable analysis. This is a standard practice in statistical analysis, since the researcher cannot include every potential factor in statistical modelling.

**Comment: 2.** There have been more interests associating paternal factors and children’s growth, for example: https://www.sciencedirect.com/science/article/pii/S0021755716304375?via%3Dihub
Please consider paternal factors, otherwise, change the title to be maternal and early life nutritional determinant factors

Response: The title now reads “Factors associated with normal linear growth among pre-school children living in better-off households: a multi-country analysis of nationally representative data”

**Comment: 3.** The authors adjusted the models for child’s age and sex in the second and final model which has raised an important issue because: among all factors, gender has been established as the most factor defining an individual’s growth that growth standards separate the growth charts based on sex. I would suggest separating the analyses based on gender or use gender as a fixed factor in the model.

Response: Adjusting for gender in the multivariable models addresses this issue. We do not think it is necessary to conduct separate analysis, using gender as a stratification variable.

**Comment: 4.** Using stunting (defined as HAZ<-2 at a time point) only as the predictor of linear growth is not wise because: 1) it does not differ stunting as growth faltering parameter with short stature, especially if not adjusted for parental height; 2) it does not reflect growth as a process, but only as a one-point measurement, 2) in paediatrics, the essential growth measurements include height, weight, adiposity (BMI as the crude/simplest measure, although is not ideal as body composition measure), and head circumference (only for children age 2 years or younger). Therefore, I would suggest complementing the analyses with catch-up/catch-down parameters (definition by ALSPAC study, BMJ 2000;320:967–71) or use height trajectories as dependent variable and then run linear mixed-models; and take into account weight-for-age, weight-for-age, and BMI-for-age scores.

Response: We thank the reviewer for this comment. It is not clear what the reviewer is inviting us to do. However, we will like to point out that this paper is not about STUNTED children but NON-STUNTED. Regarding the issue of growth as a process, these are cross sectional data, therefore, examining growth as a process may be a difficult thing to do. This is would have been possible with longitudinal data.

**Comment: 5.** The data analysis part of the methods section should explain more about the models being run, given the multiple understanding of “empirical regression models”. Is it regular regression with an emphasis on empirical use? Is it linear regression with empirical logit transformation? Is it logistic regression?

Response: We thank the reviewer for this comment. We stated in the manuscript that we employed logistic regression in the analysis.

Comments about technical issue:

**Comment: 1**. Table 1: Typos and misalignment, including column title of %/mean and SD applies for all countries but somehow missing for DRC and Ghana; “IS working” under working status ; Parity, Is breastfeeding, and number of antenatal visits should be typed in bold to ease reading the table

Response: This has been done
