# Supplementary material for: Socio-demographic factors associated with normal linear growth among pre-school children living in better-off households: A multi-country analysis of nationally representative data
Source: PLoS One. 2020 Mar 11;15(3):e0224118. doi: 10.1371/journal.pone.0224118 (PMC7065827; doi:10.1371/journal.pone.0224118)
Supplement: Attachment — Submitted filename: RESPONSES-REVIEWERS-COMMENTS-PLOSONE.docx [file pone.0224118.s002.docx]

PONE-D-19-27838R1
Factors associated with normal linear growth among pre-school children living in better-off households: a multi-country analysis of nationally representative data
PLOS ONE

Reviewer #1:

COMMENT: Thank you for addressing some of my previous comments. Variables definitions have been included and editorial issues have been thoroughly addressed. However, I’m sorry to indicate that most of my comments remain unaddressed, particularly the methodological issues. Besides, your rebuttals are largely unsatisfactory and unsupported by relevant references. It would have been better if had referenced your responses, instead of justifying just from the point of the authors’ personal experience and expertise in the field. Let the work speak for itself. At this stage, I do not recommend accepting the manuscript as it would affect the literature pool as well as the reputation of the journal. Please find my comments below.

RESPONSE: We thank the reviewer for this comment. We will ensure that every response is referenced if necessary. We are not sure about the reviewer’s assertion regarding the literature pool and the reputation of the Journal. As far as we know, this analysis, which focuses on exogenous drivers (socio-demographic factors) of child growth will contribute significantly to the literature considering the key exogenous variables (16) used in the analysis, and the analytical strategy…focusing on resources for health instead of risk factors, and positive health outcomes instead of negative (disease etc) as it is often done in the literature.

COMMENT: In my previous comment, I recommended the importance of stating the criteria for the selection of 5 countries, out of the over 50 African countries. Just for example, why Kenya is included but not Ethiopia? Your response has been “The selection of these five countries was informed by our previous work (12, 32)”. I checked these references (12, 32), both of which don’t provide any information on why data from these 5 specific countries were used. Thus, the comment still remains an important issue worthy of addressing.

RESPONSE: We thank the reviewer for this comment. We revised the sentence as follows; “The selection of the five countries was based on our previous analysis using the same countries and data (12, 32)” It is difficult to give any scientific justification for the use of the five countries and not the 50+ in Africa and over 100 across the low and middle income countries. The decision of the number of countries to use in an analysis is usually at the discretion of the researcher/analyst. Some analysts use 1 country, others 2 or more countries etc. Please refer to a few examples below. <https://bmjopen.bmj.com/content/6/9/e012615>/ [10.1017/s0021932017000505](https://doi.org/10.1017/s0021932017000505)/https://doi.org/10.1136/bmjopen-2017-017344/ <https://doi.org/10.1017/s1368980016003426/> <https://doi.org/10.1371/journal.pone.0136748> / <https://www.ncbi.nlm.nih.gov/pmc/articles/PMC4409817/> etc.

COMMENT: Line 203: You stated “Only statistically significant variables were included in the multivariable analysis”. This is wrong and a major methodological bias of the work. Selection of variables for inclusion in multivariable models is done at relaxed p-values (often at P 0.20 or 0.25); such that, variables that demonstrated P<0.20 or P<0.25 would be included in the multivariable model. Statistical significance at P<0.05 is appropriate only at the final model. Besides, I would like to put at your note that variables could also be included in multivariable models based on their theoretical (biological) relevance. Please refer to the following and other references on models building:

- Bursac Z, Gauss CH, Williams DK, Hosmer DW. Purposeful selection of variables in logistic regression. Source Code Biol Med. 2008;3:17. Published 2008 Dec 16. doi:10.1186/1751-0473-3-17

RESPONSE: We thank the reviewer for this comment. We agree with the reviewer that biologically important variables may not have to necessarily be statistical significant at the bivariate analysis stage before they are included in the multivariable analysis. And we always endeavour to acknowledge this in our publications. Refer to examples below. We have added this sentence to the manuscript “variables that were not statistically significant but considered biologically important were included in the multivariable analysis”. The type of analysis suggested in the above referenced paper are beyond the scope of our study.

<https://bmjopen.bmj.com/content/6/9/e012615> /<https://www.nature.com/articles/s41598-018-26991-4>

We however, humbly disagree with the reviewer assertion that non-significant variables should be included, whether they are considered critical by the analyst or not. This will create uninterpretable parameters and unmanageable tables. This is supported by the paper the reviewer shared: “Some methodologists suggest inclusion of all clinical and other relevant variables in the model regardless of their significance in order to control for confounding. This approach, however, can lead to numerically unstable estimates and large standard errors”

There are tens of potential drivers of child health. Since it is not possible to include all of them, there must be inclusion and exclusion criteria, and that is the purpose of the bivariate analysis…this is what is mostly done in the literature. Regarding the biases in the selection of the variables, this arises when you arbitrarily select variables without subjecting them to scientific test. In the bivariate analysis, every identified potential predictor is given the chance to be included in the multivariable analysis and the excluded variables are usually those that did not meet the inclusion criteria.

COMMENT: Earlier, I recommended including the following variables in your analysis: frequency of feeding, quality of complementary feeding, early initiation of breastfeeding, exclusive breastfeeding, vitamin A supplementation, history of infection, hygiene, sanitation, health care utilization, media exposure...etc. However, your response goes “After identifying the potential drivers (including the above referenced variables) of child growth per the literature and the UNICEF conceptual framework, we subjected them to bivariate analysis and included only statistical significant variables in the multivariable analysis. This is a standard practice in statistical analysis, since the researcher cannot include every potential factor in statistical models. Our own previous work and many others have shown that some the above referenced “proven” variables did not have a statistical significant relationship with child growth/health outcomes.

Your response above is not satisfactory for many reasons including: a) I would rather argue that the evidence in support of the importance of these variables for optimal child growth and health is more robust than the evidence against their importance. Please check meta-analysis reports on the impact of caregivers’ educational status on child health and nutritional outcomes. A lack of association in your and some others’ data doesn’t necessarily mean that the variables are of no importance in reality. There are many reasons for lack of association between two variables, particularly when using cross-sectional collected data, b) you included < 10 predictor variables, which is really not much given child growth is a multifactorial condition. Most of your variables are even sociodemographic ones like maternal age, household size, number of children in the household, which we refer to ‘distal factors’. The proximal factors, immediate determinants, are largely unaccounted. Thus, it is still better to re-run the analysis by including these variables because as far as I know, the datasets enable to do that. If you couldn’t do that for logistic reasons like table size, you need to state it as a limitation, c) There is no mention of the bi-variable analysis in the statistical method section. You also did not present any result from the bi-variable analysis. This leads me to doubt whether you have done it or not. If you have done it, please state in the method the specific tests done for the different scales.

RESPONSE: We thank the reviewer for this comment. Although we subject several variables to bivariate analysis as indicated in our early response, the focus of the paper is on exogenous variables (socio-demographic factors). After the bivariate analysis, we settled on 16 predictor variables (and not <10 variables). The reason for deciding to focus on only the exogenous variables (unfortunately we did bring this up in our earlier response) is captured in the paper as follows: “In this analysis, we included only the basic/exogenous factors (socio-demographic factors) in our empirical models. We did this because there is evidence that in examining the association between child growth outcomes and exogenous factors, the proximate factors (endogenous factors) are usually excluded to avoid biased estimation of the regression parameters of the exogenous factors (47-49). It is the case because the proximate factors are pathways through which the exogenous factors influence child nutrition (48)”

Regarding the bivariate analysis, we included this sentence to address the reviewer’s concern: “ We tested statistical significant associations of the categorical variables using chi-square, while continuous variables were tested using t-test”

COMMENT: Line 80-82. “The present study fills this gap by providing robust evidence on the critical factors associated with healthy…..” Please tone down the statement as this study doesn’t take into account many of the important child growth and nutrition enhancing variables, like meal frequency, vaccination, hygiene, micronutrient supplementation, etc.

RESPONSE: The sentence has been revised.

COMMENT: I am still not satisfied with your response to my comment on how you developed the dietary diversity score for children above 24 months. To the best of my knowledge, the infant and young child feeding practice (IYCFP) indicators guideline you followed to develop the diversity scores is aimed to be used for only under-24 months’ children. Which guideline or methodological reference is justified your use of the variable for those above 24 months? Besides, the dietary data collected by DHS for under-24 and above-24months are quite different. That is clearly stated in all questionnaires, reports and guidelines produced by DHS itself. For more, please check the following references:

- The 2018 Guide to DHS Statistics: for dietary diversity (please type 410 at your PDF page locator)

https://www.dhsprogram.com/pubs/pdf/DHSG1/Guide_to_DHS_Statistics_DHS-7.pdf

- For IYCF indicators: please check the following guidelines by WHO. 2007. Indicators for assessing infant and young child feeding practices.

Part I Definitions: http://www.who.int/nutrition/publications/infantfeeding/9789241596664/en/

Part II Measurement: http://www.who.int/nutrition/publications/infantfeeding/9789241599290/en/

- A sample Nigeria DHS 2013 report: on page 211 (on pdf viewer) or page 184 (inside the document) “The 2013 NDHS used a 24-hour recall method to collect data on infant and young child feeding for all last-born children under age 2 living with their mothers. Table 11.3……”

<https://dhsprogram.com/publications/publication-fr293-dhs-final-reports.cfm>

RESPONSE: We thank the reviewer for this comment. In the DHS final data there are two sets of dietary variables: Children dietary and mothers dietary data respectively. The child dietary variables are not disaggregated by age. Also, the dietary diversity indicator used for the IYCF is the same indicator recommended for use in children under five years. The construction of the dietary diversity score in our study was based strictly on the recommended procedures of creating the same. Below are few examples of our studies and others that used this indicator for children under 5 years.

<https://www.ncbi.nlm.nih.gov/pmc/articles/PMC6044523/#pone.0200235.ref013>/ https://doi.org/10.1017/s1368980016003426/ https://doi.org/10.1136/bmjopen-2014-005340/ https://doi.org/10.1017/ <https://doi.org/10.1371/journal.pone.0136748>

COMMENT: Your response, “…We have been using the DHS data for several years and missing data have never been one of the issues we worry about”, is also wrong. Data missing is actually one of the challenges in all DHS datasets. Cognizant of that, even reports of the DHS agency itself always include statements about missing information. You could randomly check any DHS report of any country. The tables and reports include a separate category labelled “missing”. I put here the Nigeria DHS 2013 report for your reference on this issue. Just search ‘Missing’ on your pdf and see the results. I even have checked some of the datasets and learned the data is not complete on all variables as you said. Thus, I advise you to address the issue, state the level of missing as well as whether the missing cases were significantly different from the included ones. I think it is not difficult to present at least the level of missing.

<https://dhsprogram.com/publications/publication-fr293-dhs-final-reports.cfm>

RESPONSE: We thank the reviewer for this comment. We agree with reviewer that DHS included column on missing data in their recent reports. However, missing data is not a major issue in their reports. For example, the Nigerian DHS report has an average missingness of <1% (i.e. all tables combine). This is the case with other reports. In our analysis, we did not observe missingness as a major issue, therefore we did not see the need to conduct missing data analysis e.g. explicit imputation, since it has already been taken care of by the DHS during the preparation of the data files (see statement below from the DHS). Indeed, missing data analysis is almost non-existence in the literature using the DHS data.

“One of the primary goals of The DHS Program is to produce high-quality data and make it available for analysis in a coherent and consistent form. However, national surveys in developing countries are prone to incomplete or partial reporting of responses. Additionally, complex questionnaires inevitably allow scope for inconsistent responses to be recorded for different questions. For the analyst this results in a data file containing incomplete or inconsistent data, complicating the analysis considerably. In order to avoid these problems, The DHS Program has adopted a policy of editing and imputation which results in a data file that accurately reflects the population studied and may be readily used for analysis. Primary data quality policies include”: (<https://dhsprogram.com/data/Data-Quality-and-Use.cfm>)”

COMMENT: Your response, “We feel that it will not make scientific sense to present mean estimates for categorical variables”, doesn’t make sense statistically. Mean estimates, as well as standard deviations, can be presented for both categorical and non-categorical variables. Sex is a categorical variable. You can state mean like this the mean age of females is XX year (SD), the proportion of females is XX% (SD)…. The comment refers to table 1. Thus, either include SD for all variables or remove it entirely. Alternatively, instead of SD, you could present the point estimates all variables with their corresponding 95%CI. Whichever way you do, be consistent.

RESPONSE: We thank the reviewer for this comment. We still insist that statistically, it does not make sense to estimate means and standard deviations for categorical variables. The Editor and the statisticians at PLOS ONE may also give their opinion this. We do not also think it is necessary to present point estimates and CIs when describing the characteristics of samples. It is usually a basic analysis to give a picture of the sample before the main analysis and should, therefore, not be an issue for scientific interrogation.

COMMENT: You stated, in your rebuttal, that the lack of association between education status of caregivers and the growth of children is not something unexpected. However, I would still argue that it is an unusual finding, given the known benefits of better education in improving child health and nutritional status. Thus, I still recommend you to state some of the potential reasons for the lack of the association between educational status and child growth in the Kenyan data.

RESPONSE: We thank the reviewer for this comment. This sentences have been added to address the reviewer’s concern: “However, in Kenya, maternal education did not have a significant effect on child growth outcomes. This finding is puzzling as maternal education has consistently predict child growth in the literature. Further research is needed to disentangle the possible reasons accounting for the non-significant effect of maternal schooling on child growth outcomes in Kenya”

Minor comments

COMMENT: Please also check the document thoroughly for editorials errors like following.

Line 115: “using healthy growth as the primaryr outcome variable….” Primary

RESPONSE: We thank the reviewer for this comment. This error has now been corrected

Reviewer #2:

COMMENT: In general, I have observed the improved quality of the paper and I think the content of this paper is important, especially for policy-making in the LMIC setting. However, I would encourage the authors to make further revisions, as below:

RESPONSE: We thank the reviewer for this positive comment.

1. Writing style:

COMMENT: I appreciate that there have been some improvements in the text, but I’m not sure that using the premium service of Grammarly (i.e. automatic software checking) is the best/most sufficient option for academic writing like this. I would suggest asking a native speaker from your group to thoroughly proofread the manuscript (checking the grammar accuracy, using consistently academic diction in the whole text, etc) or hiring a professional service. I’ve highlighted a few examples of mistakes that still exist in this revised manuscript:

RESPONSE: We thank the reviewer for this comment. The manuscript has been thoroughly proofread.

COMMENT: a. Line 115: “primaryr” (typo)

RESPONSE: This has now been corrected

COMMENT: b. Line 124: “The objective of this study, therefore, is…” (should be “was”)

RESPONSE: This has now been corrected

COMMENT: c. Line 333: “The results highlight….” (should be “highlighted”)

RESPONSE: Has been corrected.

COMMENT: d. Line 383-384: “Maternal work status is….” (should be “was”; “maternal work status” in some places is inconsistently written as “maternal working status”, might read better as “maternal employment status”?)

RESPONSE: This has now been corrected

COMMENT: e. Text in general: many grammar inconsistencies. You may find this link helpful: <https://www.nature.com/scitable/topicpage/effective-writing-13815989/>

RESPONSE: We thank the reviewer for providing this link. It is helpful.

COMMENT: 2. Line 31, line 279, etc: Maternal weight (measured by BMI)

This may be too meticulous, but I still don’t get it why you measure weight by BMI parameter? I might suggest writing this as “weight” (as in not taking height into account) OR adiposity (measured by BMI)

RESPONSE: We thank the reviewer for this comment. We appreciate the reviewer’s concern. However, we used weight in this context to describe the level of the BMI. e.g. BMI <18.5 kg/m^2 = is described as “underweight”; BMI =18.5-24.99 “normal weight” and so on. You cannot tell whether an individual is underweight, overweight or obese by using the absolute value from a weighing scale. You have to compute the BMI (weight in kg divided by height in m^2) before you are able to tell the weight status of the individual. This is the context within which we used the term “weight”

COMMENT: 3. I appreciate that you have changed the title to address the reviewers’ comments, but it still needs editing to address these issues: 1) after all, this study is based on African setting so it needs to mention in the title, 2) factors taken into account in the analyses were limited to maternal factors (not considering paternal influence) and infant baseline characteristics (sex and feeding, not including morbidities e.g. infections, anaemia, etc.)

RESPONSE: We Thank the reviewer for this comment. We have changed the title to read “Socio-demographic factors associated with normal linear growth among pre-school children living in better-off households: a multi-country analysis of nationally representative data”

The paternal variables such as education and employment status were not significant in the bivariate analysis. We therefore excluded them and others to make the tables manageable.

Just to add that the paper focuses of exogenous variables (socio-demographic factors). The following is a text that explains why we use this approach. “In this analysis, we included only the basic/exogenous factors (socio-demographic factors) in our empirical models. We did this because there is evidence that in examining the association between child growth outcomes and exogenous factors, the proximate factors (endogenous factors) are usually excluded to avoid biased estimation of the regression parameters of the exogenous factors (47-49). It is the case because the proximate factors are pathways through which the exogenous factors influence child nutrition (48).

4. Data analysis:

COMMENT: Line 245-253: As sex and age are the two most robust and determinant factors in postnatal growth, I am still not sure why you put both factors in the second covariates group, rather than being the fixed factor or in the first covariates group?

RESPONSE: We thank the reviewer for this comment. The sex and age are not candidate predictor variables but control variables in the this analysis. As explained above, this analysis focused more on exogenous variables. We therefore feel that controlling for them in the model should be enough.

COMMENT: 5. Although the paper is focused more on non-stunted children, growth and infections/morbidities are inseparable in LMIC setting, so please add some justification of excluding of those factors in your analyses

RESPONSE: We thank the reviewer for this comment. The following is the justification for the variables used in the analysis: “In this analysis, we included only the basic/exogenous factors (socio-demographic factors) in our empirical models. We did this because there is evidence that in examining the association between child growth outcomes and exogenous factors, the proximate factors (endogenous factors) are usually excluded to avoid biased estimation of the regression parameters of the exogenous factors (47-49). It is the case because the proximate factors are pathways through which the exogenous factors influence child nutrition (48)” Further, we also used the bivariate results to decide on which variables to include in the multivariable analysis. This decision was taken to ensure the stability of the models and make the results tables manageable

COMMENT: 6. Please pay attention to the tables and check them carefully:

a. Table 1: please use the same font size throughout; move “child-level covariates” one level down (not in the same level as %/mean and SD); “sex of child” replace with “gender” (no need to mention “child” because it’s under the child-level covariates); “IS working” should be written as “Is working”; separate “parity” and “is breastfeeding” with space so they wouldn’t seem to be part of “working status” category; etc; please check again the whole content carefully

b. Table 2-6: I would suggest writing the names of variables more clearly; for example, “working status = is working” to be “working mother”, “Sex of child = Male” to be “Male sex”, etc.

RESPONSE: This has now been corrected.
